# Viral Delivery of Small-Hairpin RNAs for Reducing Gene Expression In The Rodent Brain

**Published:** 2008

**Authors:** Amy W. Lasek, Ulrike Heberlein

**Keywords:** Alcohol and other drug disorder (AODD), genetic theory of AOD use (AODU), brain, genetics and heredity, gene knockout technology, RNA interference (RNAi), small hairpin RNAs (shRNAs), viral delivery systems, lentivirus, oligonucleotides

As described in the preceding article by Adams and Zawada (pp. 256–258), short-interfering RNA (siRNA) molecules and other related RNA molecules can be used successfully to disrupt the normal expression of specific genes in the mammalian brain—a process known as RNA interference (RNAi). However, although the strategy described by those authors has been shown to be effective, its usefulness is limited by the fact that the observed effects are only transient. For some research questions, however, it is vital that expression of a specific gene is reduced (i.e., knocked down) for a longer period of time. This article reviews a strategy to ensure long-term expression of another type of interfering RNA molecule—that is, small-hairpin RNAs (shRNAs)—through the use of viral delivery systems (i.e., vectors). Expression of these shRNAs leads to the destruction of the intermediary molecules (i.e., messenger RNA [mRNA] molecules) generated during the expression of the target gene.

## Viral Delivery of shRNA

Like the siRNAs described in the preceding article, shRNAs are short RNA molecules, containing 19 nucleotides of sequence that match the gene to be knocked down. However, in contrast to siRNAs, which consist of two separate, but complementary, RNA strands that interact with each other, shRNAs consist of a single RNA strand whose sequence is such that the two ends are complementary and therefore can interact with each other, thus forming a hairpin structure with a double-stranded stem and a single-stranded loop. Moreover, shRNA typically is not introduced directly into the cells (e.g., by transfection). Instead, the shRNA first is artificially inserted (i.e., cloned) into the genetic material (i.e., genome) of a type of virus called lentivirus.^1^ In addition, a gene encoding a fluorescent marker called green fluorescent protein (GFP) is cloned into the same virus so that any cells that are later infected by the virus easily can be identified based on their green fluorescence. The modified virus then is allowed to infect the target cells (e.g., certain brain cells).

In contrast to the genome of most organisms, which consists of DNA, the genome of lentiviruses (and some other viruses) consists of RNA. Once the virus has entered the cell, its genome is converted into DNA that then can integrate into the genome of the host cell. There, the viral genes are expressed, just like the normal cellular genes, using the cell’s own machinery. This ensures sustained expression of the viral genes, including the inserted part encoding the shRNA, thereby allowing for long-term silencing of the gene of interest.

Lasek and colleagues (2007) used such a lentiviral vector^2^ to knock-down the expression of the μ-opioid receptor (MOR) in a brain region called the ventral tegmental area (VTA). (For more information on the μ-opioid receptor and its role in alcohol and other drug dependence, see the article in this issue by Lovinger, pp. 196–214.) Various other genes also have been targeted successfully to another brain region called the nucleus accumbens using this vector. Both of these regions play a pivotal role in mediating the rewarding and reinforcing properties of alcohol and other drugs.

**Figure 12:**
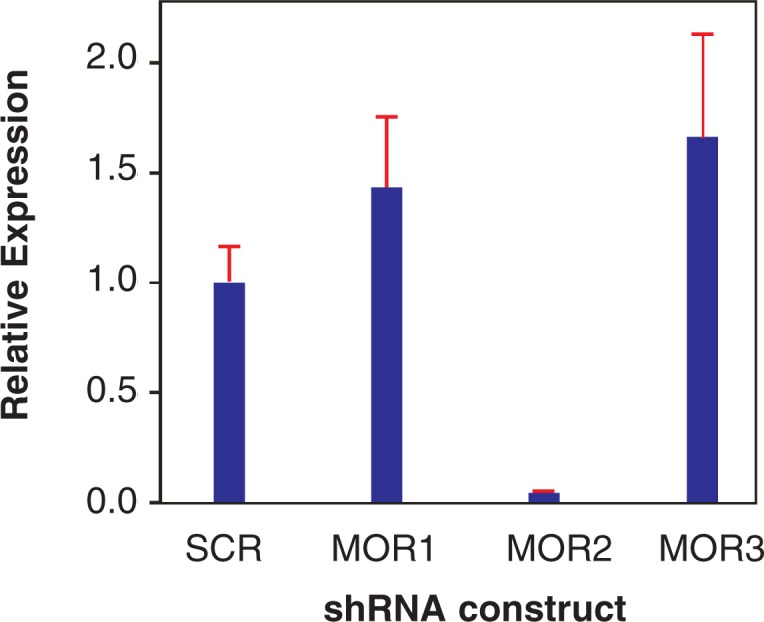
Example of the effectiveness of knock-down of expression of the μ-opioid receptor (MOR) by different small-hairpin RNAs (shRNAs). Shown is the relative level of MOR expression after introduction of three different shRNAs or a control construct (i.e., a construct containing a “scrambled” shRNA that should not target any mouse or rat gene) into cells stably expressing MOR. The results show that at 48 hours after the introduction of the three constructs and the control, construct MOR2 is most effective, essentially shutting down the cell’s MOR production.

An essential step in using this approach to reduce or eliminate expression of a specific gene is the design of suitable shRNAs. In general, shRNAs are designed based on a variety of criteria developed by Reynolds and colleagues (2004). To simplify the process, researchers can use publicly accessible Web sites to design shRNAs for their specific experiments, such as the Thermo Scientific Dharmacon siDesign Center (available at: http://www.dharmacon.com/DesignCenter/DesignCenterPage.aspx) or the siRNA Web site at the Whitehead Institute (available at: http://jura.wi.mit.edu/bioc/siRNAext/). For each gene, three candidate target sequences of 19 nucleotides are chosen. Then, nucleotide chains (i.e., oligonucleotides) are synthesized in the laboratory that contain complementary 19-nucleotide target sequences with the loop sequence inserted between the two complementary sequences (so that they can form the characteristic stem-loop structure of the shRNA) and a signal sequence that stops expression of the shRNA at the right site. Once these oligonucleotides have been introduced into the lentivirus vector, they are first tested in cultured cells to determine if and how effectively they can knock down target gene expression (see figure 12).

**Figure 13:**
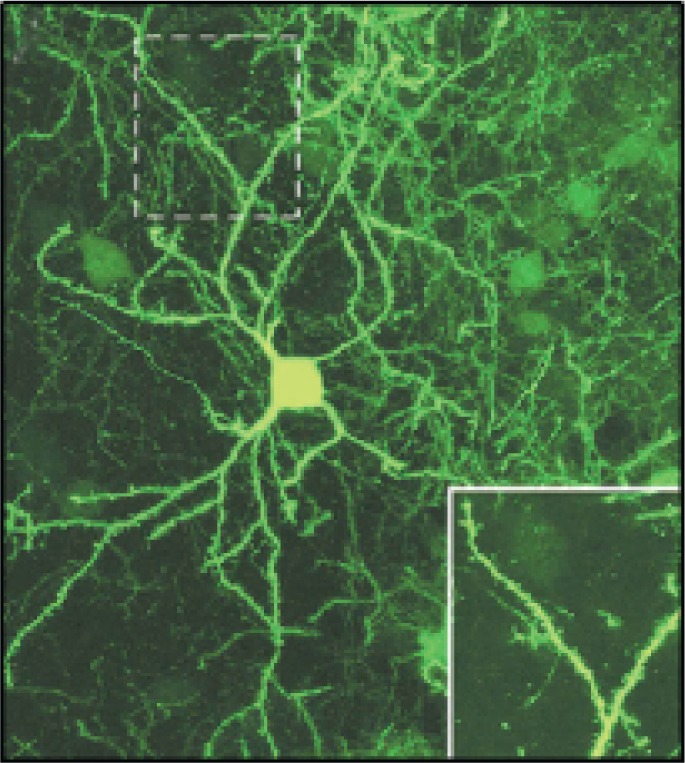
Example of a neuron located in the mouse nucleus accumbens that has been infected by a lentivirus carrying a small-hairpin RNA (shRNA). The success of the infection is demonstrated by the green fluorescence of the cell, which stems from a marker gene (i.e., green fluorescent protein [GFP]) that was introduced into the cell via the same virus as the shRNA. The image was taken 2 weeks after the infection. The inset shows a higher magnification view of the dashed rectangle, illustrating GFP expression even in the tiny extensions protruding from the neuron.

**Figure 14:**
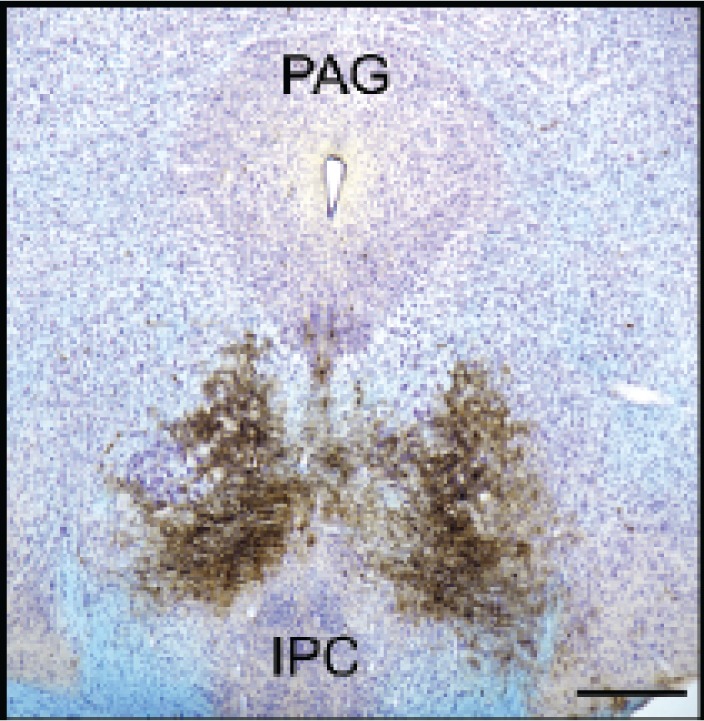
Expression of a gene introduced into the mouse brain using a lentivirus delivery system at 6 weeks after the infection. Brown spots indicated the expression of the marker gene, green fluorescent protein (GFP), which was introduced by lentivirus infection of the ventral tegmental area on both sides of the brain. The image demonstrates that the lentivirus system likely results in stable, long-term expression not only of the introduced marker gene but also of the shRNA. NOTES: IPC = interpeduncular nucleus; PAG = periaqueductal gray area.

After efficient shRNA sequences have been identified, the corresponding modified virus is prepared in larger quantities (e.g., using commercially available kits) and purified. It then is injected into the brain region of interest, and knock-down of the target gene can be assessed approximately 2 weeks later (see figure 13). Initial analyses then serve to determine the time frame of the gene knock-down (i.e., when exactly and for how long knock-down is strongest). Once the timeframe of gene knock-down in the living animal (i.e., in vivo) has been determined, additional animals are infected with the modified lentivirus and their behavior tested using appropriate tests. When these behavioral analyses are completed, the site and extent of infection are determined by staining the appropriate brain sections or treating them with immune molecules (i.e., antibodies) that make cells expressing the GFP marker gene visible (see figure 14).

## Conclusion

Studies have found that shRNAs expressed from lentiviral vectors result in sustained knock-down of expression of the gene of interest. This technology can be used to examine the role of a particular gene in specific brain regions and has been useful for studying behaviors related to drugs of abuse, including alcohol.

## Footnotes

1 During the cloning process, the shRNA is placed under the control of a regulatory element (i.e., a promoter) that ensures constant (i.e., constitutive) gene expression.

2 This lentiviral vector originally was developed by Rubinson and colleagues (2003); it now is commercially available from The American Type Culture Collection.
